# Chemical reaction network knowledge graphs: the OntoRXN ontology

**DOI:** 10.1186/s13321-022-00610-x

**Published:** 2022-05-30

**Authors:** Diego Garay-Ruiz, Carles Bo

**Affiliations:** 1 Institute of Chemical Research of Catalonia (ICIQ), The Barcelona Institute of Science and Technology, Av. Països Catalans 16, 43007 Tarragona, Spain; 2 Departament de Química Física i Inorgànica, Universitat Rovira i Virgili, Marcel . lí Domingo s/n, 43007 Tarragona, Spain

**Keywords:** Ontologies, Reaction networks, Semantics, Reactivity

## Abstract

**Abstract:**

The organization and management of large amounts of data has become a major point in almost all areas of human knowledge. In this context, semantic approaches propose a structure for the target data, defining ontologies that state the types of entities on a certain field and how these entities are interrelated. In this work, we introduce OntoRXN, a novel ontology describing the reaction networks constructed from computational chemistry calculations. Under our paradigm, these networks are handled as undirected graphs, without assuming any traversal direction. From there, we propose a core class structure including reaction steps, network stages, chemical species, and the lower-level entities for the individual computational calculations. These individual calculations are founded on the OntoCompChem ontology and on the ioChem-BD database, where information is parsed and stored in CML format. OntoRXN is introduced through several examples in which knowledge graphs based on the ontology are generated for different chemical systems available on ioChem-BD. Finally, the resulting knowledge graphs are explored through SPARQL queries, illustrating the power of the semantic approach to standardize the analysis of intricate datasets and to simplify the development of complex workflows.

**Graphical Abstract:**

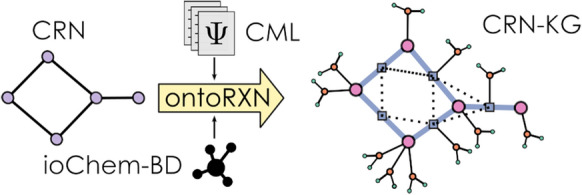

**Supplementary Information:**

The online version contains supplementary material available at 10.1186/s13321-022-00610-x.

## Introduction

**Fig. 1:**
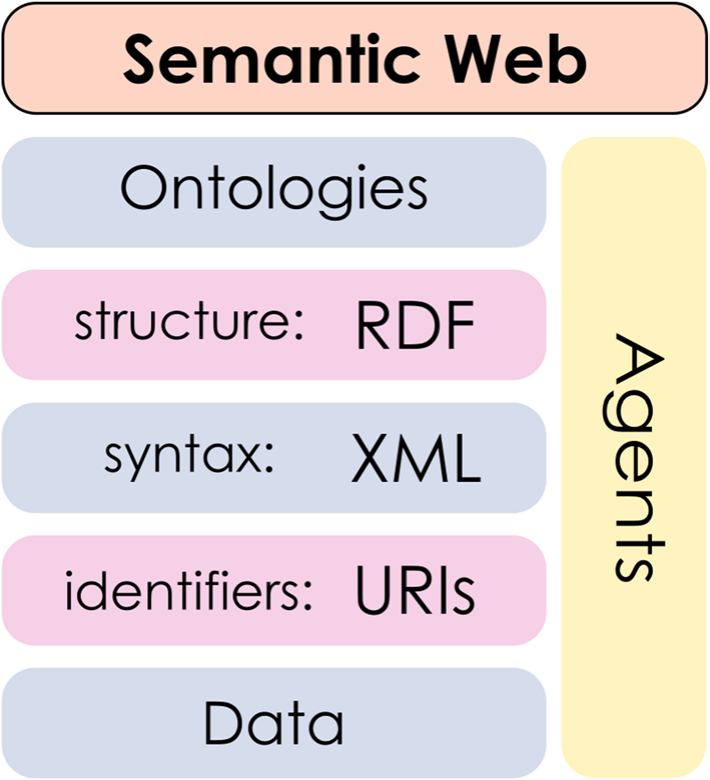
Base elements for the Semantic Web proposal, from Berners-Lee [4], depicted as stacked layers

The development of sensible, shareable and scalable data models has become an essential asset in nearly every area of science and knowledge. Data-driven approaches are rapidly increasing their impact, in such a way that the focal point is not as much the storage, but instead the ways in which data can be retrieved, explored and utilized [1–3]. To reach this, more sophisticated (“smarter”) approaches to data organization are necessary. Among these, we can highlight the *Semantic Web*, proposed by Tim Berners-Lee [4] in 2001, whose basic building blocks are outlined in Fig. 1. The goal of the Semantic Web is to add logic and structure to the data in the World Wide Web, permitting the application of reasoning schemes. Given that the Web is nothing else than a collection of linked information, these principles may then be applied to any other specific body of knowledge. The information in the Semantic Web can be fetched by smart *agents* capable of making *inferences* based on the relationships between the entities, potentially answering complex questions about the data. In this context, data is identified by URIs [5] (Uniform Resource Identifiers), which provide a consistent and web-conforming notation scheme for each element. These elements are formatted as tags, using the XML [6] (eXtended Markup Language) format. Then, the RDF [7] (Resource Description Framework) data model provides meaning to the tags, structuring the information through the assertion of triples of the form “subject–predicate–object”. Finally, once all entities have been introduced, it remains to define the *relationships* between them: in other words, to build an ontology.

Ontologies propose *classes* to characterize the different elements existing in a certain domain of knowledge, and then define how these classes relate between themselves through *properties*, effectively building a representational vocabulary of the target domain [8], also known as a *taxonomy*. In this sense, ontologies provide a standardization of knowledge, explicitly defining common terms and structures that can be shared and reutilized between different communities. Besides, these definitions can be used as templates for the data and metadata required to express the entities in a field of knowledge (e.g., user input forms). The term *knowledge graph* (KG), sometimes expressed as knowledge *base*, is used for datasets that have been expressed and categorized under the class structure of an ontology: the corresponding class members are denoted as *individuals* of the KG. Currently, the OWL (Web Ontology Language) [9] format, an ontology-oriented extension of RDF, is the language of choice for expressing ontologies and knowledge graphs.

Moreso, this kind of well-structured information allows to easily connect data coming from different sources, following the paradigm of Linked Data [10]. While specific areas of knowledge require specific ontologies, these (and their corresponding KGs) may be bridged by stating equivalencies between their common elements. Regarding scientific data, ontologies have been widely adopted in biology and biomedicine [11–13], but are not yet as common in other fields like Chemistry. Among the existing chemical ontologies, for which a recent review was carried out by Pachl et al. [14], we may highlight ChEBI, for molecules of biological interest [15], CHMO, for the formalization of methods in experimental chemistry [16], RXNO, for conceptualizing chemical reactions [17] or OntoKin [18], for kinetic studies on mechanisms. There is a remarkable multiscale nature in the current chemical ontology ecosystem, from very low-level descriptions of phenomena, like in the reaction representations developed by Shankar and collaborators [19–21], describing up to the electron shells of atoms, to developments oriented to full laboratory automations as proposed by Kraft et al. [22] or the more general information-driven approach of the CHEMINF ontology for cheminformatics [23, 24].

Computational chemistry seems particularly suited for this kind of approach, due to the digital nature of all generated information. Indeed, some proposals on the matter have already been done, such as Gainesville Core [25], a set of basic definitions aiming for “a complete description of a typical Computational Chemistry experiment”. Gainesville Core has then been used as the starting point for larger developments such as OntoCompChem [26], which was recently combined with OntoKin [27] to connect computational studies with a more general description of chemical kinetics. Nevertheless, as the use of these ontologies has not yet been extended along the community, many aspects remain to be developed. While defining a complete ontology including every aspect of computational chemistry would be a daunting task, developing smaller specific ontologies and connecting them afterwards shows as a more feasible goal.

**Fig. 2:**
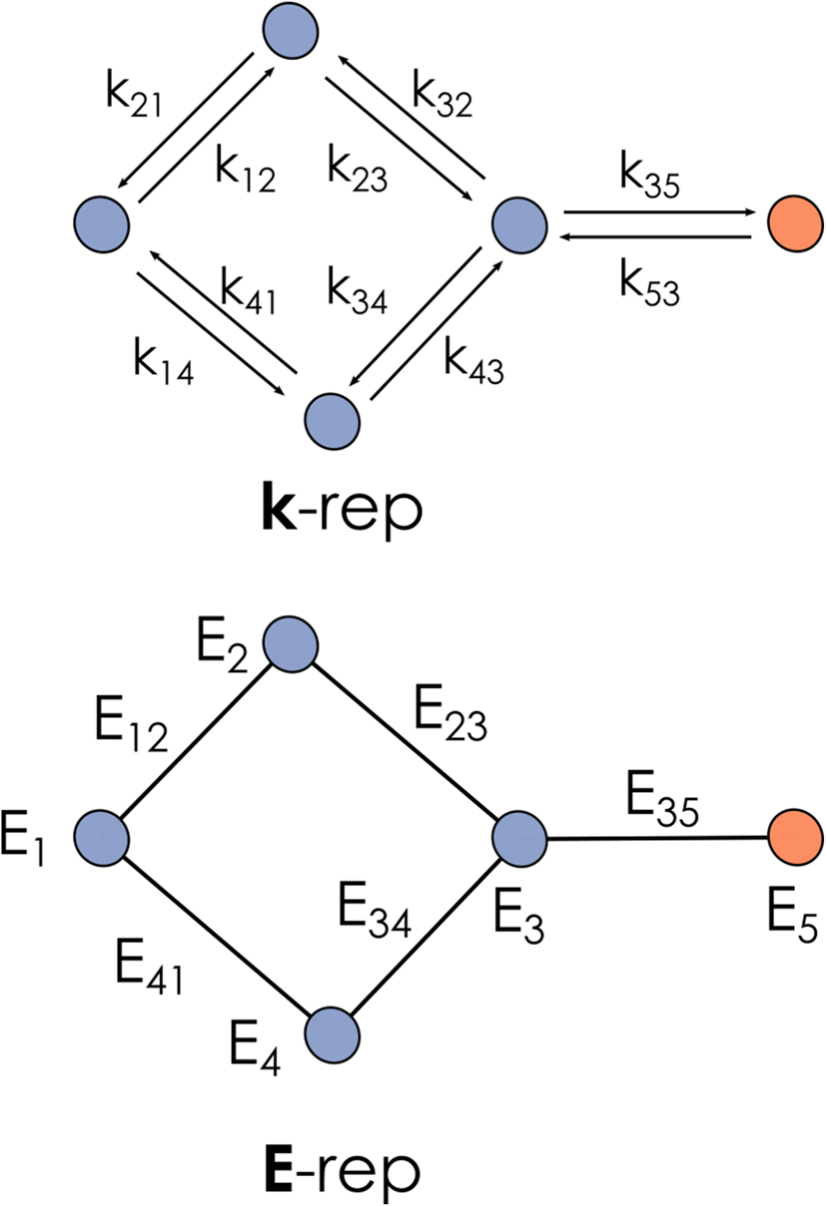
Schematic depiction of the directed k-representation (above) and the undirected E-representation (below) initially defined in the context of the energy span model and proposed here as building block for OntoRXN. Here, $\hbox {k}_{{ij}}$ symbols represent rate constants, $E_{i}$ node energies and $E_{ij}$, edge energies

In this line, one area of application where a semantic-based organization could be useful is the study of reaction mechanisms and chemical networks. Recent efforts by our group have been devoted to the development of novel open-source tools for the treatment and processing of reaction networks through graph-based approaches: amk-tools [28] and gTOFfee [29]. The latter is an application of the energy span model (ESM) developed by Sebastian Kozuch and collaborators [30, 31] extended to manage reaction networks as *undirected* graphs [32]. In this sense, no forward or reverse direction is assumed to construct the network: the chemical flow comes from the *exergonicity* of the embedded reactions. Consequently, there is a switch from the traditional **k**-representation, based on reverse and forward rate constants, to the **E**-representation [33], undirected and based only on energies (Fig. 2). This change of paradigm permits a much more natural pairing with computational chemistry results, which provide energy and not rate constants as a main output, and simplifies the final graph structures.

The concept of reaction mechanism introduced in previous ontologies, such as OntoKin, was built on the classical rate-constant-based representation. This approach, while perfectly valid, lacks the immediate matching with computational data from the **E-**representation. Therefore, we took this undirected description of reaction networks as the foundation for a new ontology for computationally characterized reaction mechanisms: OntoRXN. We aim to directly connect this ontology with the ioChem-BD database [34, 35], a central piece for our data management workflow. ioChem-BD is a service which parses the outputs obtained from many common computational chemistry codes, such as Gaussian, ADF, VASP, MOLCAS, ORCA..., to store the results in an unified CML [36–39] (Chemical Markup Language) format. The information contained in those CML files can be visualized and accessed through the ioChem-BD platform, allowing users to easily share information. Reaction networks can be also defined inside the platform, providing meaning and structure to the stored data, in line with the principles of the Semantic Web. While other projects tackling the semantic-based publication of computational chemistry results proposed the definition of new formats (e.g. CSX [40]) to overcome some limitations of CML, we believe that the connection with the already established ioChem-BD database justifies the direct use of CML. In this sense, the development of OntoRXN supposes another step forward in the standardization of information, presenting knowledge graphs as a standard format combining all the information for a given reaction mechanism: the computational results from the CML files and the network structure interlinking the calculations, which embeds the chemical knowledge about the system.

Following this idea, the main guidelines for the design of OntoRXN were:

- Apply the **E**-representation: networks as fully undirected graphs.
- Use the information available on the CML files from ioChem-BD: readily available and already properly tagged.
- Aggregate individual calculations into molecule sets: chemical reactions and catalytic cycles do not usually refer to a single molecule per step, but instead group several species that have to be taken into account to preserve the number of atoms across the network.

While there is an evident discrepancy in “directedness” between our OntoRXN proposal and the pre-existing solutions (OntoKin + OntoCompChem [27]), both descriptions could be linked altogether, as the **k**- and **E**-representations are indeed equivalent. Activation free energies can be transformed into rate constants through the Eyring equation, eventually converting our undirected, energy-based graph to a directed, rate-constant-based one. Under the ontology paradigm, this will be achieved through *agents* tailored to traverse the network encoded in the KG and assign proper directionality.

## Ontology development

**Fig. 3:**
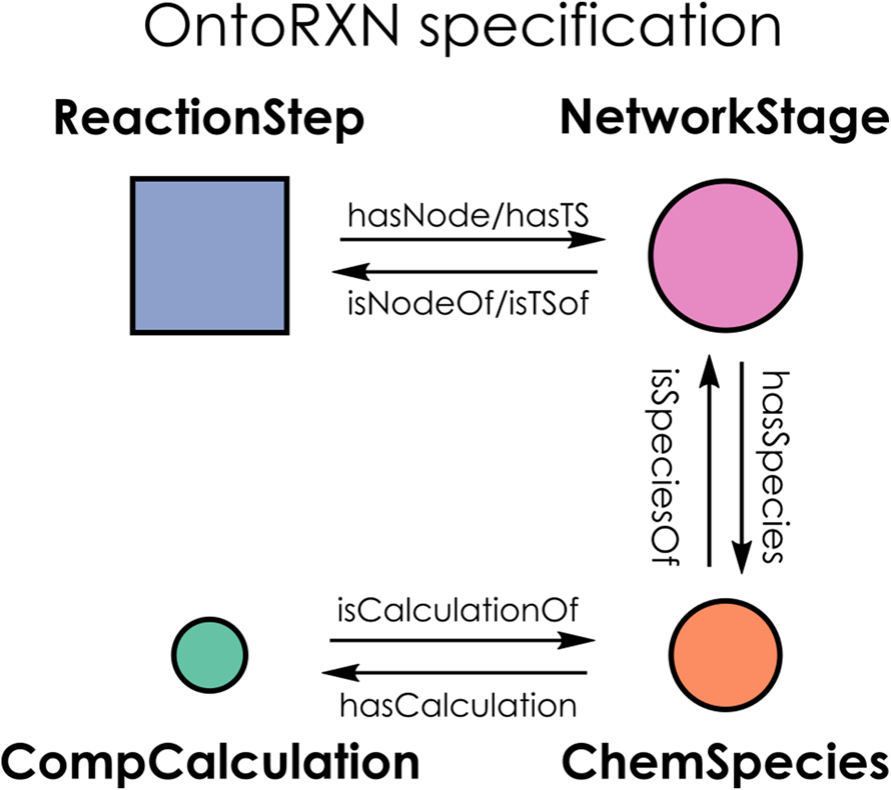
Core class structure (topology) for OntoRXN, specifying the four main classes and the properties interlinking them

Following the previous guidelines, we propose four core classes on OntoRXN, whose basic relationships are depicted in Fig. 3.

- CompCalculation. Storage of all computational data, as taken from the ioChem-BD preprocessed CML file.
- ChemSpecies. Representation of individual structures for molecules and transition states. Several CompCalculation objects could be mapped to an unique ChemSpecies (e.g. calculations at different levels of theory, with different solvation models, etc).
- NetworkStage. Set of structures (as *ChemSpecies*) that have to be considered together in a given point of the reaction network or catalytic cycle. All *NetworkStage* objects in a given knowledge graph should have a consistent number of atoms, so properties such as relative energies can be properly computed across the graph. Both intermediates and transition states along the mechanism would be expressed as *NetworkStage* objects, which may include several additional reactants or products that appear in any other part of the cycle or reaction set.
- ReactionStep. Set of stages comprising two linked *intermediates* and (possibly) the corresponding *transition state*, all expressed as *NetworkStage* objects. The undirected nature of the networks under the E-representation implies that no directionality-related properties need to be defined when instantiating either a *ReactionStep* or a *NetworkStage*. The connection between different steps is simply determined by shared intermediates (aka *nodes*), so two given steps will be linked whenever they comprise a common *NetworkStage*.

To connect OntoRXN with the ecosystem of existing ontologies, we employed OntoCompChem [26] as a base for the *CompCalculation* class, doing the necessary extensions over the OntoCompChem core. Apart from the definition of additional properties (present in the CML specifications from ioChem-BD but not yet defined in the original ontology), we considered a refactorization of OntoCompChem’s class structure. The current version of OntoCompChem has a main *GaussianCalculation* class in its topology, grouping subclasses related to different versions of the program, but does not consider yet any generalization to calculations performed with any other program. As ioChem-BD already handles the parsing step, converting all output files to CML format, our protocol should integrate this independence from the code employed to carry out the calculations. To overcome this issue on the ontology side, we added a *BaseCalculation* superclass to properly extend OntoCompChem to handle the results from different programs, with the idea of adding child classes for the programs whose outputs are supported by ioChem-BD.

On the other hand, the *ReactionStep* class, which refers to the elementary reaction steps in a given reaction network, shall also include information about the *type* of transformation to which it corresponds. Elementary molecular processes are collected in the MOP (Molecular Process Ontology) ontology, distributed with the RXNO ontology for chemical reactions [17]. Thus, we defined a *hasReactionType* property for ReactionStep entities, with the general *molecular process* class in MOP as its range: then, the types of elementary reactions in MOP can be directly mapped to the steps defined in OntoRXN. However, reaction type labelling assumes a direction for the reaction, a concept which is not on our ontology proposal. Thus, reaction type assignment should involve *pairs* of types for a given step (e.g. oxidation/reduction, fragmentation/association...), with specific agents dealing with the KG being the ones to handle the assignment of a direction to each member of the pair.

In principle, this basic 4-class structure, together with the connection with the more general OntoCompChem ontology, should be enough to start instantiating knowledge graphs for different example networks. The use of real data to generate the corresponding KGs will allow to find possible points of further development of the ontology and possible connections with pre-existing ones, following the common iterative development process of ontologies.

### Knowledge graph generation from ioChem-BD

**Fig. 4:**
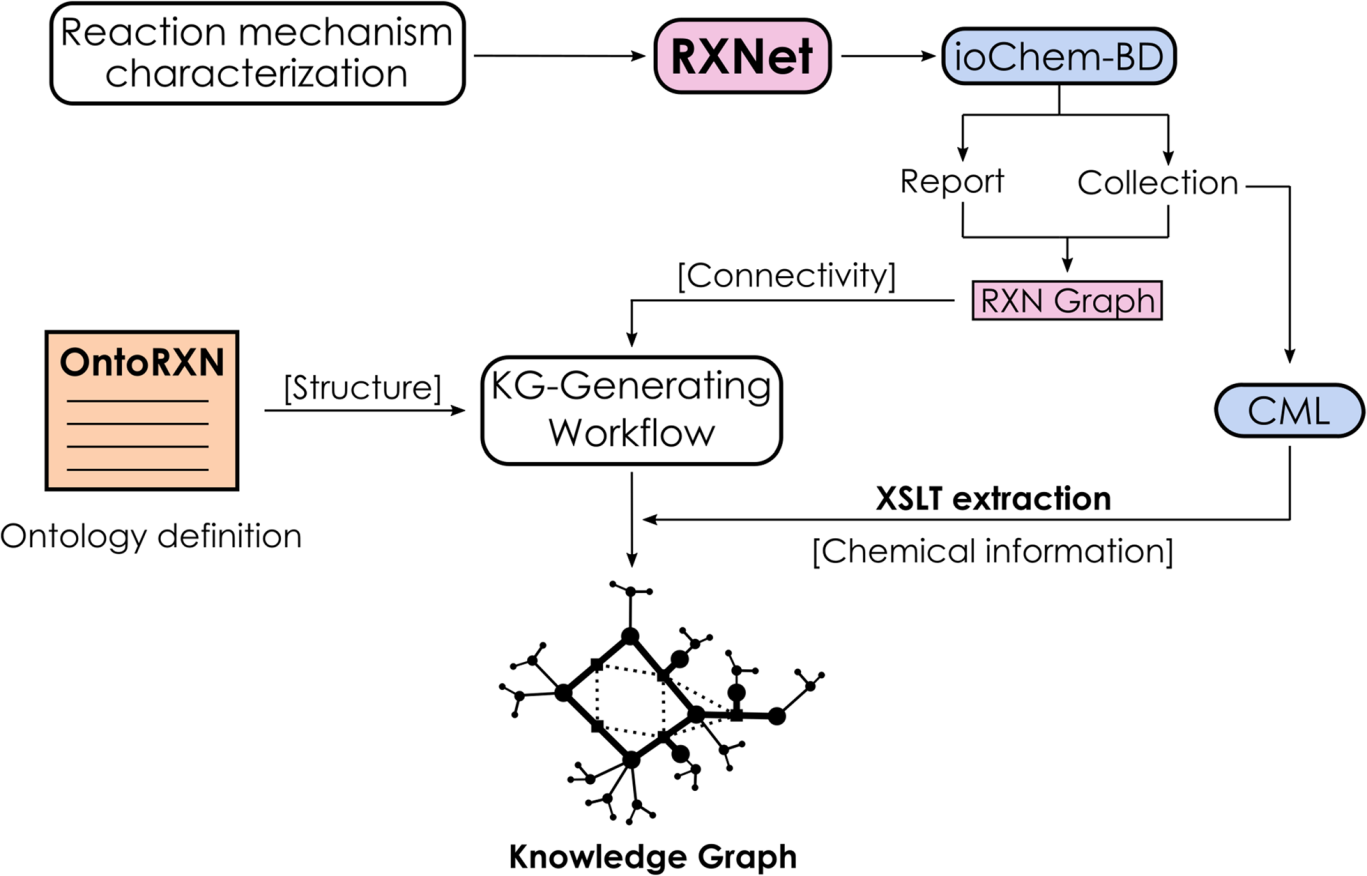
Workflow scheme for ioChem-BD/OntoRXN-based knowledge graph generation

As mentioned before, recent additions to ioChem-BD allow for the construction of reaction network graphs from reaction energy profiles built in the platform [28]. These reaction networks are an ideal starting point for the generation of KGs: all information about the individual calculations can be easily fetched from the CML files, and structured according to the corresponding network topology. Eventually, it would be possible to add the knowledge-graph-building machinery as an additional module in the platform, thus allowing the user to painlessly create OntoRXN-compliant graphs.

As a first approach to such an implementation, we built a Python interface, provided as the *ontorxn-tools* module distributed with the ontology, to manage the complete pipeline from fetching and processing the data in ioChem-BD to the generation of the knowledge graph, as summarized in Fig. 4.

The process starts by characterizing the target reaction mechanism, either manually searching all intermediates and transition states or employing automated search tools. The predicted mechanism defines a reaction network (RXNet), which can then be uploaded to ioChem-BD. There, the individual calculations are stored in a *collection*, while the structure of the graph is defined as a *report* where the network is described through a set of individual profiles including all species and connections across the graph. In this way, we obtain the connectivity of the network as this graph in DOT format, while the calculation information can be obtained by querying the corresponding report through ioChem-BD’s REST API.

Later, *ontorxn-tools* is used to parse the graph and the associated CML files. Technical details about the implementation of this step of the protocol is available in the Additional file 1: Section S3. After parsing, the library generates the final knowledge graphs, structured through the set of classes and properties defined in OntoRXN and fed from the information in the CML files.

### Semantic querying of knowledge graphs

One of the main goals of this semantic approach to information is, as stated in the Introduction, the ability to formulate and answer complex questions from data. Eventually, a fully ontology-organized body of knowledge would allow the user to make arbitrarily complicated queries targetting widely different properties of the system under study. For example, in the case of Chemistry, a query may bring together aspects about energies from computational calculations, properties of the solvents employed to carry out a given synthesis, prices of reagents... with all of these factors being defined in different, but interrelated, chemical ontologies. While such a degree of development and interconnection may still be quite far at the time being, the semantic querying of smaller knowledge graphs, like the reaction networks described through OntoRXN, does already provide a powerful tool to simplify the analysis of complex systems.

Because ontologies depend on the RDF data model, the SPARQL [41] query language developed for RDF databases arises as the tool of choice for this kind of tasks. Like RDF, SPARQL is also based on the statement subject/predicate/object *triples*, but in the query language some of the elements in the triple can indeed be *variables*. The application of these triple patterns to an RDF graph provides a RDF subgraph including only the triples matched by the query, which can be employed either to effecively add new statements to the main graph (CONSTRUCT query) or to isolate these subsets to answer questions (SELECT query). Several SPARQL endpoints to semantically-organized chemical databases, such as RHEA [42] or the Integrated Database of Small Molecules [43] (IDSM), have been proposed.

Moreover, combining SPARQL querying with scripting, we can develop powerful workflows (or, following the original nomenclature from the Semantic Web proposal, *agents*) that can retrieve the information encoded in the knowledge graphs to generate plots, do further calculations and post-processing, or generate structured inputs for additional simulations. To demonstrate the versatility of this approach, we will consider three examples of computational mechanistic studies on different systems, whose reaction networks have been transformed to knowledge graphs and processed by specific agents tackling points of interest from the original studies.

## Applications

### Mapping the knowledge graph

**Fig. 5:**
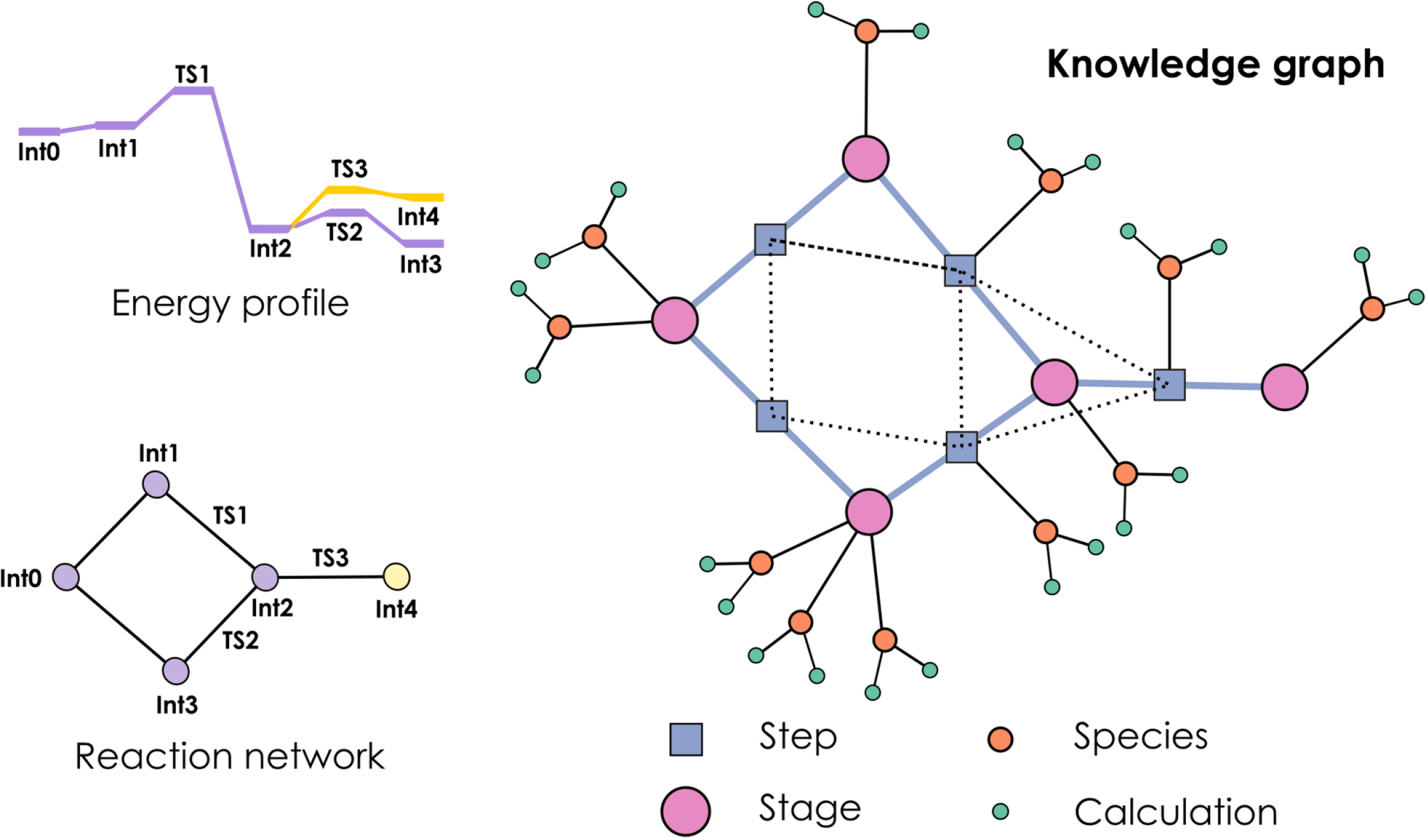
Schematic depiction of possible mechanism depictions for peroxyformate decomposition. Above left, reaction energy profile comprising two routes. Below left, basic reaction network structure with nodes as intermediates and edges as transition states. Right, simplified OntoRXN-based knowledge graph, with hierarchical step > stage > species > calculation structure

**Fig. 6:**
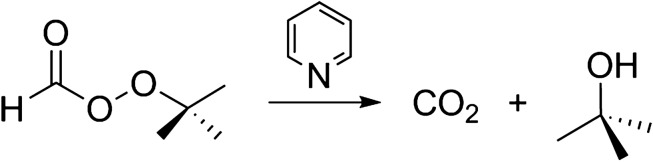
Peroxyformate decomposition reaction, producing carbon dioxide and tert-butanol in the presence of pyridine as an organocatalyst

We will be using our recent mechanistic study on the decomposition of tert-butyl peroxyformate [44], in Fig. 6, to showcase the generation of knowledge graphs from the OntoRXN ontology. This system provided us with a relatively simple mechanism (which can be encoded in a small reaction network), whose assorted calculations were already available at ioChem-BD, thus supposing an ideal test case for both OntoRXN and *ontorxn-tools*. The corresponding representations for this mechanism (energy profile, reaction network and knowledge graph) are shown in Fig. 5.

The knowledge graph depiction in Fig. 5 highlights the additional information provided by the highly explicit ontology-based approach compared with the plain reaction network graph. While the basic network structure is somehow preserved in the *NetworkStage* objects (pink circles), the KG makes clear how some stages group several different molecules (as *ChemSpecies*, orange circles), and how these molecules might have several assorted *CompCalculation* entities (green circles). This study, indeed, considered the recalculation of the reaction mechanism in a wide variety of implicit solvents, which can be naturally expressed by the one-to-many mapping between species and calculations defined in OntoRXN. For compactness, the elemental steps (*ReactionStep* entities) are depicted here as blue squares in the middle of the lines connecting the stages for their reaction intermediates. These steps are interconnected by dashed lines, which highlight another layer of connectivity along the network.

It should be recalled that Fig. 5 is only a simplified representation of the KG, without including any of the chemical descriptors obtained from the actual calculations. These magnitudes are mapped to the individual *CompCalculation* entities, and defined as objects containing not only the value of the descriptor itself but also its units, when applicable. While at the moment we have focused on a small core property subset as a demonstration, including only electronic energy, Gibbs free energy, geometry, method, basis set, vibrational frequencies and InChI string descriptors, the ontology and the accompanying XSL stylesheet should be expanded in the future, eventually targetting all properties captured in ioChem-BD.

Once we have defined our entire reaction network as a knowledge graph, we can easily extract information on the system through SPARQL queries and process it as needed. Once we have defined our structured KG, it is straightforward to write a query to fetch a given individual property from every *CompCalculation* entity and then group the results by the corresponding *NetworkStages* the calculations (via the *ChemSpecies* they belong to) are linked to. This approach produces tables mapping every stage in the reaction network to properties such as their total energy, for instance. From this basic proposal, queries can be further refined, filtering and grouping the results as needed.

For example, for the process of peroxyformate decomposition we were interested in how the Gibbs free energy of the rate-determining step, from the reactants to the first transition state, varied depending on the solvent. To do this, we would need to know the energies of the corresponding intermediates and transition states for every solvent in the network. Therefore, our question to the knowledge graph could be: *What are the electronic and free energies for every stage in the network for every different solvent?*. If we “translated” this question from English to SPARQL (with a couple of additional modifications), we get would get the query in Additional file 1: Listing S6. This query produces a table listing all unique combinations of stages and solvents, combining the energies from the appropiate calculations across the complex KG containing results for 29 total implicit solvents and 300 individual calculations. From there, we may easily obtain plots comparing activation energies with the polarity of the solvent, as done in the initial mechanistic study [44] (see Additional file 1, where Fig. S1 reproduces Figure 2 from that original study).

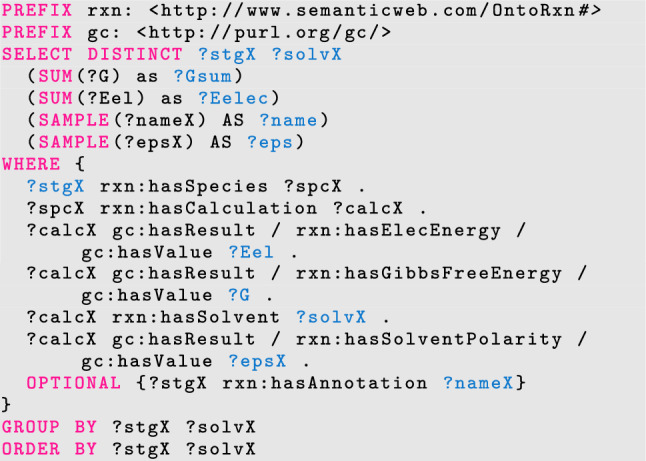



Building on this protocol, analogous queries can be easily built to fetch any other property of interest across the knowledge graph, providing an unified interface to all the information generated by the calculations and encoded in the reaction network. The strong organization of the resulting tables or results allows for a very simple post-processing to, for example, generate plots like the one in Additional file 1: Fig. S1.

### Complex reaction networks

**Fig. 7:**
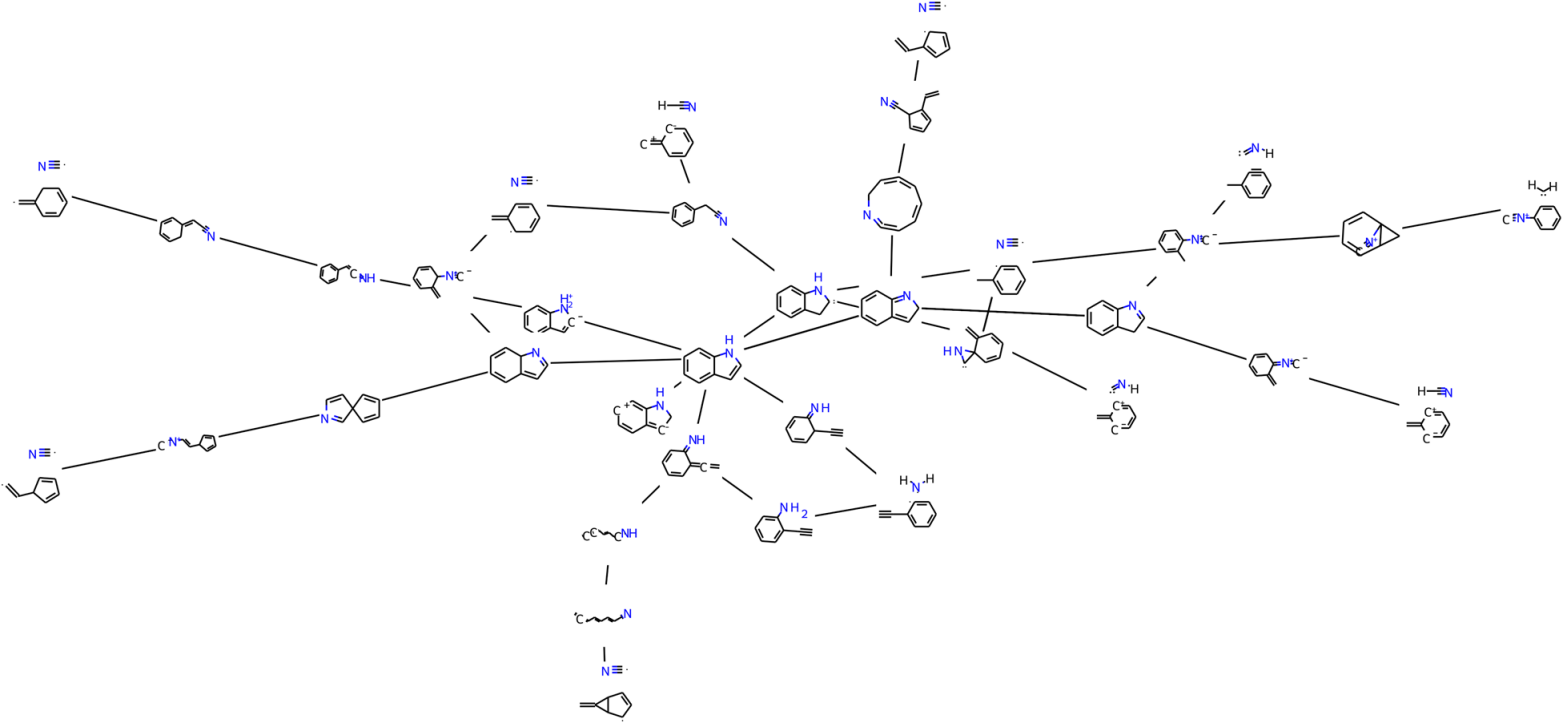
Reaction network graphs for indole decomposition, with molecular depictions for every node taken from InChI strings

While the previous example relied on a “hand-made” mechanistic characterization, there is a growing interest in the automation of mechanistic searches, employing either chemical heuristics or reactive molecular simulations. Irrespectively of the details of how the exploration is carried out, these tools end up producing some kind of *reaction network* as output, revealing the connectivity between the different species characterized along the search. These likely quite complex networks provide an immediate target for the application and development of our ontology.

As we mentioned before, we recently developed the amk-tools [28] library to process and visualize the networks discovered by AutoMeKin [45–47], a flexible, open-source program for automated mechanism predictions. The amk-tools toolkit allows not only to easily process the obtained networks, but also to directly upload the network topology and the accompanying calculations to the ioChem-BD platform in a fully automated manner. Therefore, the ioChem-BD-based protocol to build KGs can be directly used for AutoMeKin results, wrapping up the overall workflow.

As a target system for this AutoMeKin–ioChem-BD–OntoRXN pipeline, we considered the decomposition of indole, which we recently studied to showcase the capabilities of the amk-tools package, and which like the peroxyformate example was already available on ioChem-BD [28]. The resulting KG shows a total of 69 stages and 40 steps, many more than in the previous example, but also has a simpler 1:1 mapping between calculations and species, demonstrating the different kinds of complexity that we may encounter when treating chemical systems.

In this more intricate network, a question of interest might be how many times a given fragment appears across different stages of the knowledge graph: the corresponding query and the tabular results are provided in the Additional file 1: Listing S1 and Table S1. Cyanide radical is the most common species, appearing in a total of six stages, followed by HCN and HNC, participating in two stages each. The rest of species in the network, in contrast, are only matched to unique stages.

To wrap everything up, it is also possible to build a query to recover the original reaction network graph used to build the connectivity on the KG (see Additional file 1 for additional details). While this process might seem redundant at first (build the KG from the reaction network, then generate the very same reaction network from that KG), it allows us to effectively employ our OntoRXN-based knowledge graphs as a standard format to share the networks together with all required calculation data. In this way, KGs can be easily integrated in existing workflows relying on “traditional” reaction networks (e.g. gTOFfee input, depiction of reaction mechanisms, graph-based profile searches...), with the advantage of having a simple and robust way to feed more information into these simple networks. For example, we can map the InChIs identifying all the species belonging to each node in the network, which we can process to obtain 2D molecular representations (using the RDKit [48] library) that can be embedded in the network depiction (Fig. 7).

### Utilization of KGs in complex simulation workflows

**Fig. 8:**
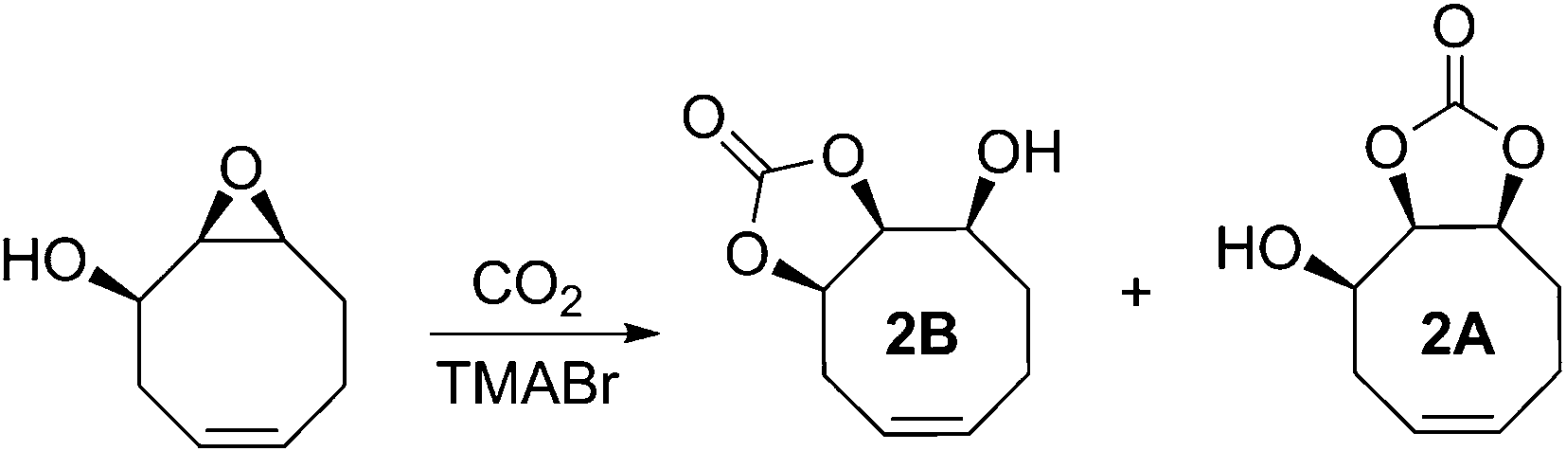
$\hbox {CO}_{{2}}$ fixation reaction over cyclooctene epoxy alcohol derivative, including the two main diastereoisomeric cyclic carbonate products 2A and 2B

While on the previous examples we have focused on utilizing the knowledge graph for the direct *analysis* of the data it contains, it can also be employed as an standard format to generate input for other calculation tools, selectively extracting the calculation properties required in each case. This approach can be especially valuable for building complex workflows and automating tasks, minimizing the need for manual user input. As an example of this, we built a microkinetic model from the knowledge graph of the reaction network for a $\hbox {CO}_{{2}}$ fixation process whose mechanism was studied by our group [49] (Fig. 8). In that article, we employed microkinetic modeling to demonstrate the thermodynamic control of the reaction, reproducing with good accuracy the experimental values of conversion and selectivity.

Assuming all processes to be reversible, we can build a model by defining all the reactions encoded in the network, computing their barriers in both possible directions. From these barriers, we can use the Eyring equation to compute the pertinent rate constants. With all processes being reversible, we do not need to assign any direction on network traversal, as the chemical flow will be marked by the own simulation.

Additionally, when applying microkinetic models to homogeneous systems in solution [50], we shall take into account the change in the reference state for Gibbs free energies, going from 1.0 atm in the gas phase (as present in standard output files) to 1.0 M in solution. The reference temperature may also be modified, improving the match with experimental conditions: here, calculations were done at the standard temperature of 25 $^\circ$C, while the working temperature for the reaction was 80 $^\circ$C. Recomputing these energy corrections does only require a trivial recalculation of partition functions, employing the standard formulas on statistical thermodynamics. Once again, the knowledge graph structure simplifies the task of extracting the relevant magnitudes required for these partition function recalculations: namely, electronic energy, vibrational frequencies, moments of inertia, molecular mass and symmetry number.

**Fig. 9:**
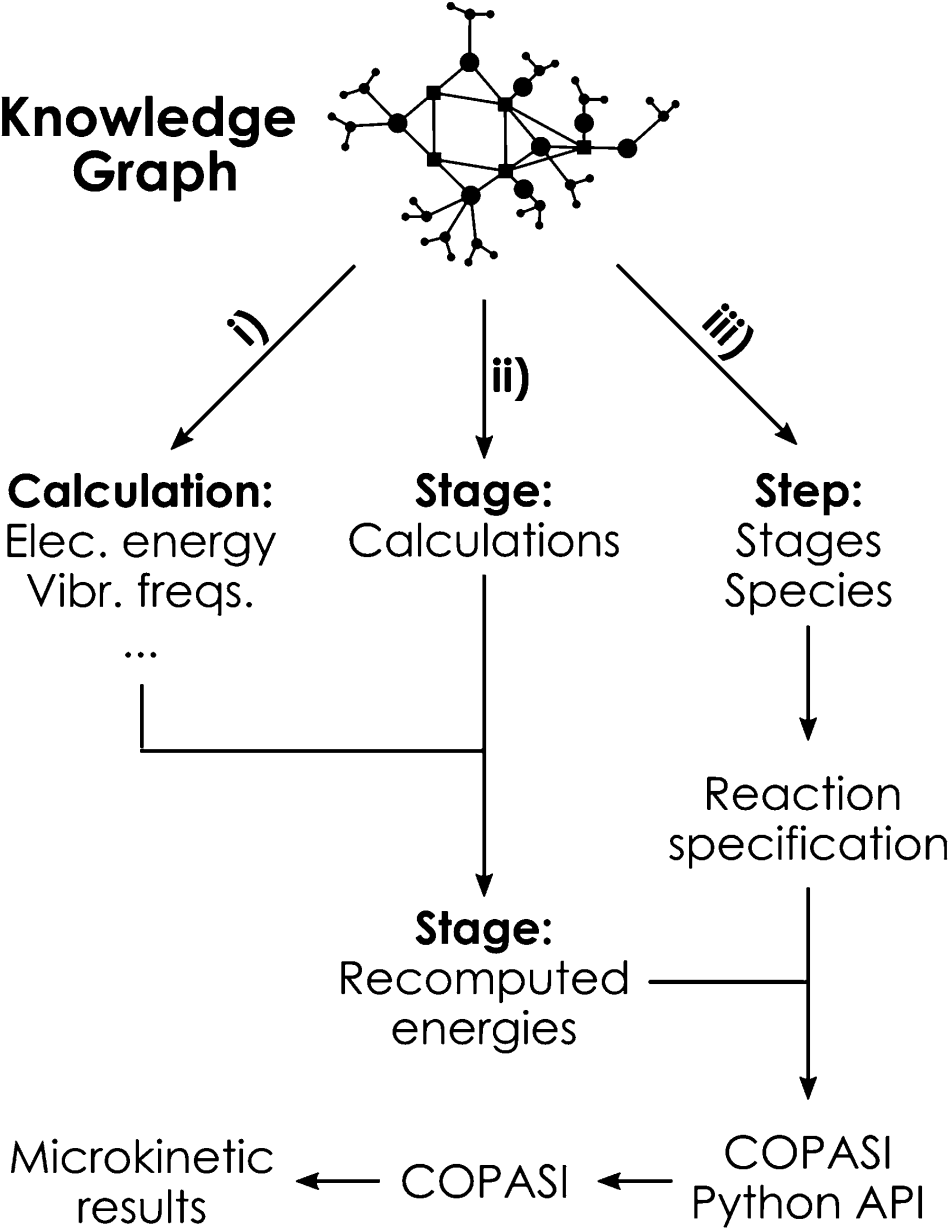
Schematic depiction of the querying workflow to set up a microkinetic model in COPASI from the knowledge graph

In terms of working with the knowledge graph, we need three queries (Fig. 9) to (i) fetch all properties for partition function recalculation, (ii) match stages with their calculations, and (iii) process the unique reactions encoded in the graph. From (i) it is possible to recompute Gibbs free energies at the requested pressure and temperature, while (ii) allows to match stages with all the calculations they comprise, so individual energies can be summed obtaining stage energies. The unique reactions from (iii) come directly from the *ReactionStep* entities in the graph: for every step, we only need to select the two interlinked *NetworkStages*, and go along the *ChemSpecies* that they contain. The reaction is then defined by stating that the species on one of the stages are transformed to the species on the other, removing later on the species that appear on both sides. Precomputed stage energies are finally mapped to the steps, so the relative forward and reverse barriers required for the microkinetic model can be computed.

**Fig. 10:**
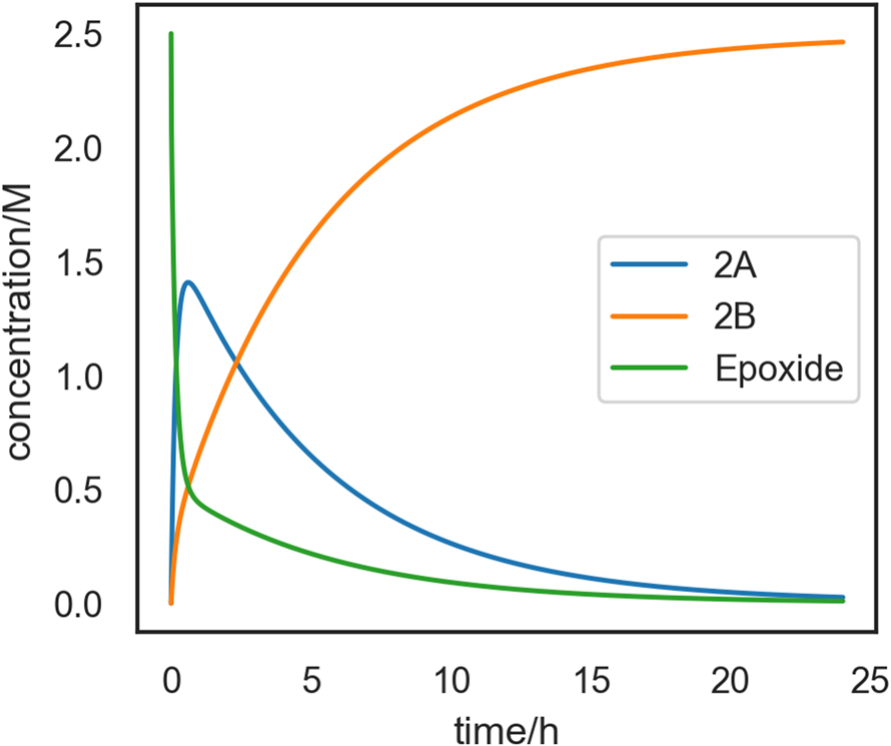
Concentration vs. time plot for $\hbox {CO}_{{2}}$ fixation on a epoxy alcohol cyclooctene derivative [49], at T = 353.15 K and assuming 1.0 M reference state for all species

From the data fetched from the knowledge graph, we employed COPASI [51] and its Python API to generate the final microkinetic model and run the corresponding time course simulation programatically, passing the external parameters (reagent concentrations, temperature, simulation time) to obtain the profile in Fig. 10.

The main strength of this kind of workflows based on knowledge graphs is their transferrability: the current simulation protocol might in principle be applied to any other reaction network, as the KG provides an already standardized format containing all required information. Consequently, task automation becomes easier, avoiding the likely time-consuming steps of parsing and organizing the information from raw outputs.

## Conclusions and future work

Our main goal throughout this manuscript was to present our novel ontology-based approach for reaction networks, aiming at the standardization of this kind of entities in an unified format containing both network topology and individual calculation results. Up to now, this proof of concept consists of:

- Definition and development of the core ontology structure for OntoRXN.
- Development of the knowledge graph instantiation agents (ontorxn-tools) linked to the ioChem-BD platform.
- Development of agents for the post-processing and utilization of OntoRXN-based KGs as a standardized format for reaction networks, integrating them into pipelines for data analysis or further simulation.

From there, several development areas and possible applications for the ontology and the derived knowledge graphs arise naturally.

- Extension of the ontology, incorporating new properties and classes for the fields already available on the CML files generated by ioChem-BD. Simultaneously to ontology growth, the XSL stylesheets employed for querying these CML files shall also be expanded.
- Generation of a database of computed catalytic cycles expressed as knowledge graphs.
- Development of smarter agents for the instantiation and extension of knowledge graphs, introducing features such as the automated identification of the reaction types defined in the Molecular Process Ontology.
- Identification and development of other possible connecting points from OntoRXN to other relevant chemical ontologies.

## Supplementary Information

**Additional file 1.** Additional details on the applications, example for regeneration of reaction network graphs, and description of the OntoRXN-Tools.

## Data Availability

The OntoRXN ontology [52] and the ontorxn-tools [53] library are both available at GitLab. Additionally, a custom RDF database and SPARQL endpoint has been set up as a web service [54], containing the three knowledge graphs used as demonstrations throughout the manuscript (peroxyformate decomposition, indole decomposition, $\hbox {CO}_{{2}}$ fixation), including all the SPARQL queries that were employed as examples. This service is based on the Blazegraph [55] RDF database, adapting the default workbench instance as the user frontend, which allows to explore the three KGs and to run SPARQL queries against them.
