# Supplementary material for: Chemical reaction network knowledge graphs: the OntoRXN ontology
Source: J Cheminform. 2022 May 30;14:29. doi: 10.1186/s13321-022-00610-x (PMC9153116; doi:10.1186/s13321-022-00610-x)
Supplement: Supplementary file 1 — Additional file 1. Additional details on the applications, example for regeneration of reaction network graphs, and description of the OntoRXN-Tools. [file 13321_2022_610_MOESM1_ESM.pdf]

RESEARCH

Supporting Information for: Chemical Reaction Network Knowledge Graphs: the OntoRXN Ontology

Diego Garay-Ruiz[1,2*] and Carles Bo[1,2*]

*Correspondence: dgaray@iciq.es; cbo@iciq.cat
[1]Institute of Chemical Research of Catalonia (ICIQ), The Barcelona Institute of Science and Technology Av. Països Catalans 16, 43007, Tarragona, Spain
[2]Departament de Química Física i Inorgànica, Universitat Rovira i Virgili, Marcel·lí Domingo s/n, 43007 Tarragona, Spain
Full list of author information is available at the end of the article

Contents

1 Additional details on applications **1**
1.1 Peroxyformate decomposition 1
1.2 Indole decomposition 1
1.3 CO_2 fixation on cyclooctene epoxy alcohol 2

2 Regeneration of reaction network graphs **3**

3 Description of OntoRXN-Tools **4**
3.1 Input graph processing 4
3.2 Ontology management 5
3.3 Knowledge graph instantiation 6

1 Additional details on applications

1.1 Peroxyformate decomposition

Results from the query in Listing 1 in the main text were employed to build the plot in Figure S1.

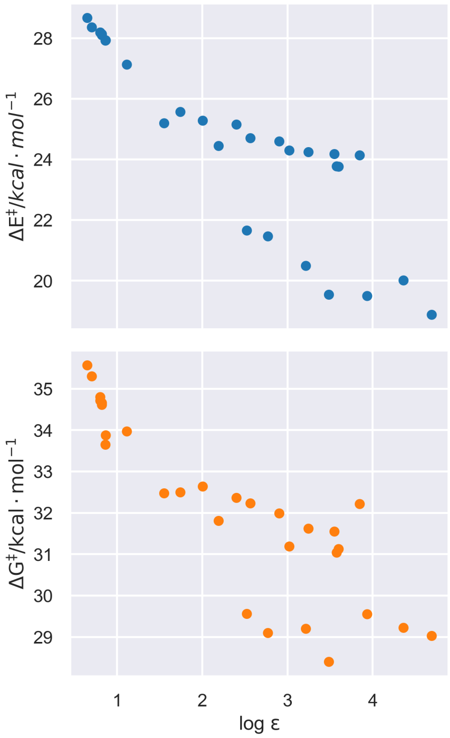

Figure S1: Barrier heights (electronic and free energy), in kcal·mol^{-1}, for peroxyformate decomposition against the logarithm of the solvent polarity, as in our previous study[1], extracted directly from the knowledge graph.

1.2 Indole decomposition

```
PREFIX rxn: <http://www.semanticweb.com/OntoRxn#>
SELECT DISTINCT ?spcX (COUNT(?stgX) AS ?Ncount)
  (SAMPLE(?labX) AS ?lab)
  (GROUP_CONCAT(?nameX) AS ?stages)
WHERE {
  ?stgX rxn:hasSpecies ?spcX .
  ?spcX rxn:hasCalculation ?calcX .
  ?calcX rxn:hasAnnotation ?labelX .
  BIND(STRBEFORE(?labelX,';') AS ?labX)
  OPTIONAL{?stgX rxn:hasAnnotation ?nameX}
}
GROUP BY ?spcX
ORDER BY DESC(?Ncount)
LIMIT 5
```

Listing S1: SPARQL query example to query the number of stages in which each species in the system appears and their corresponding names, returning only the five top results ordered by occurrence number.

Listing S1, once again, shows the lexical simplicity of the core queries, with a couple of additional SPARQL statements to directly simplify some of the queried strings at the query level. In *ontorxn-tools*, the names of the individual species are stored through the **hasAnnotation** property, together with the calculation order and ID in the underlying ioChem-BD report. The string manipulation in the query (via STRBEFORE) does already select the name part only, simplifying the obtained table. The results of this query are collected in S1.

Frag.	No. stages	Sel. stages
CN	6	PR342 PR313 PR315 PR136 PR320 PR409
HCN	2	PR3 PR101
HNC	2	PR120 PR278
PR155	1	PR155
CH2	1	PR155

Table S1: Number of occurrences per network stage of the most common fragments in the indole network and corresponding stage names selecting the top five results only.

1.3 CO_2 fixation on cyclooctene epoxy alcohol

The three queries required to build the microkinetic model (schematized in Figure 7 of the main text) are collected in Listings S2, S3 and S4.

```
PREFIX rxn: <http://www.semanticweb.com/OntoRxn#>
PREFIX gc: <http://purl.org/gc/>
SELECT DISTINCT ?calcX ?Eel ?molmass ?moi ?symmnumb ?freqlist
WHERE {
  ?calcX gc:hasResult / rxn:hasElecEnergy / gc:hasValue ?Eel .
  OPTIONAL{?calcX gc:hasResult / rxn:hasMolMass / gc:hasValue ?molmass} .
  OPTIONAL{?calcX gc:hasResult / rxn:hasMomentInertia / gc:hasValue ?moi} .
  OPTIONAL{?calcX gc:hasResult / rxn:hasFreqList / gc:hasValue ?freqlist} .
  OPTIONAL{?calcX rxn:hasSymmetryNumber ?symmnumb}
  }
ORDER BY ?calcX
```

Listing S2: SPARQL query to fetch descriptors for partition function and thermodynamic magnitude recalculation, including electronic energy, molecular mass, moment of inertia, symmetry number and vibrational frequencies, for every *CompCalculation* object in the KG.

```
  PREFIX rxn: <http://www.semanticweb.com/OntoRxn#>
  PREFIX gc: <http://purl.org/gc/>
  SELECT DISTINCT ?stgX (SAMPLE(?nameX) AS ?name)
    (GROUP_CONCAT(?calcX ; separator=';') AS ?calcList)
  WHERE {
    ?stgX rxn:hasSpecies ?spcX .
    ?spcX rxn:hasCalculation ?calcX .
    OPTIONAL {?stgX rxn:hasAnnotation ?nameX}
  }
  GROUP BY ?stgX
  ORDER BY ?stgX
```

Listing S3: SPARQL query to map *NetworkStage* entities to their corresponding calculations, assuming 1:1 mapping between species and calculations.

```
PREFIX rxn: <http://www.semanticweb.com/OntoRxn#>
PREFIX gc: <http://purl.org/gc/>
SELECT DISTINCT ?stepX ?stgX ?stgTS
        (GROUP_CONCAT(?spcName ; separator='+') as ?spcNode)
WHERE {
  ?stepX rxn:hasNode ?stgX .
  ?stgX rxn:hasSpecies ?spcX .
  ?spcX rxn:hasCalculation ?calcX .
  ?calcX rxn:hasAnnotation ?noteX .
  BIND(STRBEFORE(?noteX,';') AS ?spcName) .
  ?stepX rxn:hasTS ?stgTS
}
GROUP BY ?stepX ?stgX ?stgTS
ORDER BY ?stepX
```

Listing S4: SPARQL query to fetch reaction specifications from the KG, mapping every *ReactionStep* to the corresponding stages and the *ChemSpecies* belonging to them.

2 Regeneration of reaction network graphs

As mentioned in the main text, the possibility of recovering the basic network connectivity from the knowledge graph is important regarding the utilization of KGs as a standard network expression format.

This underlying network structure is encoded through both the *ReactionStep* and the *NetworkStage* classes, with every **step** mapping to an edge in the original network and being linked through the *hasNode* property to two nodes defined as **stages** in the KG. The transition state, if present, will also be linked to the step via the *hasTS* property.

```
PREFIX rxn: <http://www.semanticweb.com/OntoRxn#>
SELECT ?stepX (GROUP_CONCAT(?stgX) as ?stgL)
  (SAMPLE(?stgY) AS ?stgTS)
WHERE {
?stepX rxn:hasNode ?stgX .
OPTIONAL {?stepX rxn:hasTS ?stgY}
}
GROUP BY ?stepX
```

Listing S5: SPARQL query example to extract basic network connectivity from the knowledge graph, locating the two nodes connected by each step and, if present, thecorresponding transition state.

Node	Node	TS
rxn:STAGE_932-stg-4	rxn:STAGE_932-stg-0	rxn:STAGE_932-stg-5
rxn:STAGE_932-stg-2	rxn:STAGE_932-stg-0	rxn:STAGE_932-stg-6
rxn:STAGE_932-stg-0	rxn:STAGE_932-stg-1	rxn:STAGE_932-stg-7
rxn:STAGE_932-stg-2	rxn:STAGE_932-stg-3	None
rxn:STAGE_932-stg-3	rxn:STAGE_932-stg-4	None

Table S2: Results of the connectivity query on Listing S5 for the peroxyformate example network[1], with the stage identifiers of pairwise-connected nodes and the corresponding TS (if applicable)

The simple query on Listing S5 provides directly the connectivity of the network encoded in the knowledge graph, including the necessary identifiers for all NetworkStages: an example is provided in Table S2.

From there, any property might be added through additional queries that map stage identifiers to the requested values, such as energies. In this sense, and returning again to the peroxyformate network, we can depict the reaction mechanism with the Gibbs free energies computed in a selection of solvents (Figure S2).

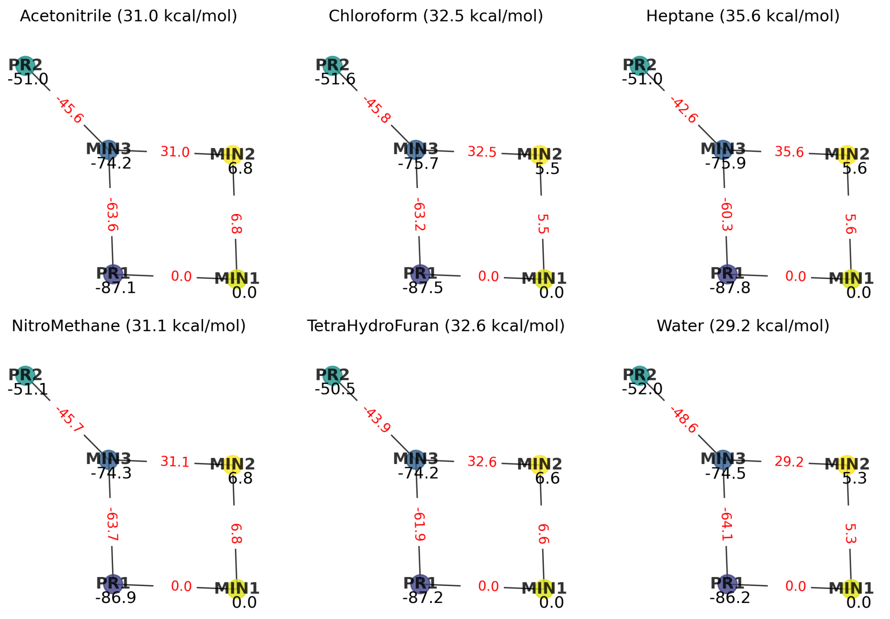

Figure S2: Reaction network graphs for peroxyformate decomposition in a selection of six solvents (acetonitrile, chloroform, heptane, nitromethane, tetrahydrofuran and water). The overall free energy reaction barrier for each solvent is given between parenthesis after each solvent name.

3 Description of OntoRXN-Tools

The *ontorxn-tools* module provides a Python interface to handle the connection between the calculations stored in the ioChem-BD platform, as CML files, and the deployment of the final knowledge graphs based on the OntoRXN ontology. Additionally, the *py-iochem* package, also provided in the repository, contains submodules to manage ioChem-BD's reports, CML files and graphs. While the outline of the main workflow was already introduced in the main text, we think that some additional details on the design and functioning of the library may be valuable for the reader.

3.1 Input graph processing

The expected input format for graphs in *ontorxn-tools* is the DOT format generated by the Create module of ioChem-BD, with management of this graph on the Python side being done through the NetworkX library[2]. So, the DOT input is read into a NetworkX.Graph object (and split into several objects if the input graph has

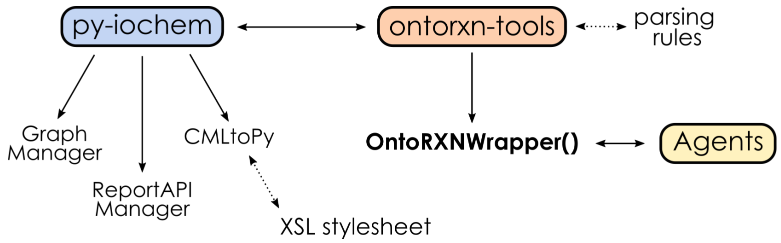

Figure S3: Schematic package structure for ontorxn-tools and py-iochem: dotted arrows connect modules with external files required for the main workflow.

several disconnected subgraphs). Together with this graph structure, the ID of the corresponding report (reportId) in ioChem must should be provided, as this entity will be queried through the REST API to fetch the calculation IDs (calcId) of each unique calculation in the report. From there, the same REST API can be employed to fetch and download the CML files so they can be accessed locally. In the future, this direct connection with reports and calcIds shall facilitate the eventual integration of KG generation inside the ioChem-BD platform.

3.2 Ontology management

The *OntoRXNWrapper* class allows to simplify general I/O, generation and transformation of OntoRXN-based knowledge graphs. Both the owlready2[3] and RDFLib[4] libraries are employed to generate and handle these knowledge graphs, switching from the OWL-compliant representation of the former and the more general RDF representation of the latter. Our wrapper class allows to seamlessly switch between the two descriptions, from the owlready2.Ontology object to its corresponding world expressed as a RDFLib.Graph, with the first being used for general ontology access and modification and the second for querying. Moreso, the wrapper also handles the direct transformation of this RDFLib.Graph to a NetworkX.Graph object preserving the complete structure of the KG, for its visualization or processing.

3.3 Knowledge graph instantiation

First of all, each calculation fetched from the report is parsed and assigned a *CompCalculation* object. This is achieved through eXtended Stylesheet Language Transformations (XSLT), where specific stylesheets are defined to select a subset of fields present in the CML file. This approach is also applied in other modules of the ioChem-BD platform, to present the information in the CML files to the user in the web interface, converting CML to HTML. Here, we do not get HTML output, but a complex string containing key-value pairs that can be then transformed to a Python dictionary, to effectively map field names to the corresponding ontology properties. These parsed properties are then fed onto the *CompCalculation* entities in the ontology, with the property name/CML field assignments depending on the specific property. For simple properties, a set of mapping rules (available in `resources/parsing_rules.dat`, easily modifiable and extendable) relates ontology property, CML field, data type and units. For more complex entities such as geometry, which implies the instantiation of individual entities for the molecule and its atoms, the definition is hard-coded inside the *calc_instantiation()* function.

Then, the name that the current calculation has in the report definition is checked: if it has been encountered for the first time, a new *ChemSpecies* is generated and mapped for the calculation. Else, as another species would have been already defined, the calculation is linked to that previous entity, as one-to-many mappings between species and calculations are possible (as in the peroxyformate example). The calculation name was selected as the key variable here for simplicity and flexibility, but other magnitudes such as the InChI might also be used in this step.

At this point, once all species and calculations are defined, the network structure can be introduced. First, all nodes (intermediates) in the input graph are iterated through, defining a *NetworkStage* for each one. As the graph contains the formulas used in Create to define the reaction network, these stages can be mapped to the corresponding *ChemSpecies* entities, thus properly linking the ontology. After the nodes, there is an iteration along edges, which will define the *ReactionStep* entities: as node stages are already defined, the steps can be immediately linked to the corresponding node pairs. Moreover, for these edges corresponding to a TS structure, its *NetworkStage* is also defined and mapped, effectively finishing the core knowledge graph.

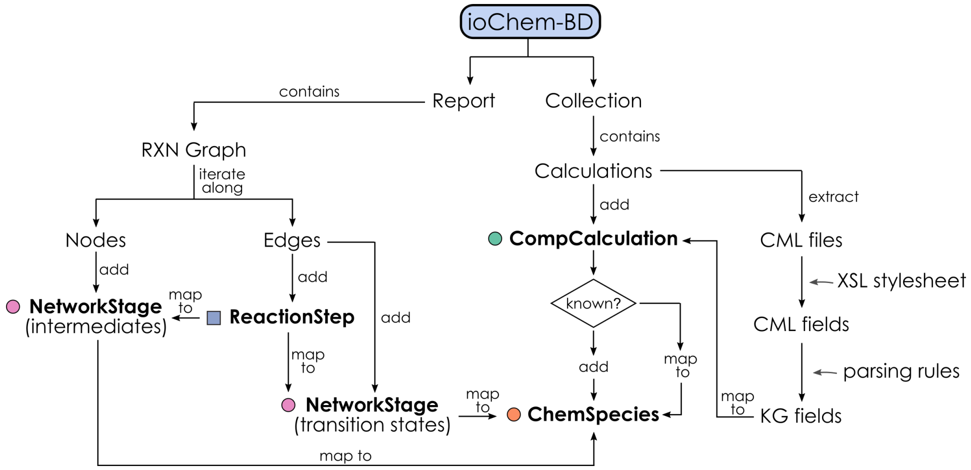

Figure S4: Detailed workflow scheme for ioChem-BD/OntoRXN-based knowledge graph generation.

Some relationships in OntoRXN cannot be properly expressed in terms of OWL relationships: for example, the connection (via the *isConnectedWith* property) between steps sharing a common stage. These situations are handled through some built-in SPARQL CONSTRUCT queries that infer the required relationships and add the facts directly on the ontology, completing the knowledge graph.

```
PREFIX rxn: <http://www.semanticweb.com/OntoRxn#>
PREFIX gc: <http://purl.org/gc/>
SELECT DISTINCT ?stgX ?spcX ?solvX (SUM(?G) as ?Gsum)
  (SUM(?Eel) as ?Eelec) ?nameX ?eps
WHERE {
  ?stgX rxn:hasSpecies ?spcX .
  ?spcX rxn:hasCalculation ?calcX .
  ?calcX gc:hasResult / rxn:hasElecEnergy / gc:hasValue ?Eel .
  ?calcX gc:hasResult / rxn:hasGibbsFreeEnergy / gc:hasValue ?G .
  ?calcX rxn:hasSolvent ?solvX .
  ?calcX gc:hasResult / rxn:hasSolventPolarity / gc:hasValue ?eps .
  OPTIONAL {?stgX rxn:hasAnnotation ?nameX}
}
GROUP BY ?stgX ?solvX
ORDER BY ?stgX ?solvX
```

Listing S6: SPARQL query example to collect electronic energy, Gibbs free energy, solvent and solvent dielectric constant for all stages in the KG.

Author details
[1]Institute of Chemical Research of Catalonia (ICIQ), The Barcelona Institute of Science and Technology Av. Països Catalans 16, 43007, Tarragona, Spain. [2]Departament de Química Física i Inorgànica, Universitat Rovira i Virgili, Marcel·lí Domingo s/n, 43007 Tarragona, Spain.

References

1. Garay-Ruiz, D., Bo, C.: Rationalizing the Mechanism of Peroxyformate Decomposition: Computational Insights To Understand Solvent Influence. Chem. - A Eur. J. **27**(45), 11618–11626 (2021). doi:10.1002/chem.202100755
2. Hagberg, A.A., Schult, D.A., Swart, P.J.: Exploring network structure, dynamics, and function using NetworkX. In: 7th Python Sci. Conf. (SciPy 2008), pp. 11–15 (2008)
3. Lamy, J.B.: Owlready: Ontology-oriented programming in Python with automatic classification and high level constructs for biomedical ontologies. Artif. Intell. Med. **80**, 11–28 (2017). doi:10.1016/j.artmed.2017.07.002
4. RDFLib: RDFLib (2022). https://github.com/RDFLib/rdflib Accessed 2022-02-16
